# The pro-inflammatory phenotype of the human non-classical monocyte subset is attributed to senescence

**DOI:** 10.1038/s41419-018-0327-1

**Published:** 2018-02-15

**Authors:** Siew-Min Ong, Eva Hadadi, Truong-Minh Dang, Wei-Hseun Yeap, Crystal Tze-Ying Tan, Tze-Pin Ng, Anis Larbi, Siew-Cheng Wong

**Affiliations:** 1 Singapore Immunology Network (SIgN), Agency for Science, Technology and Research (A*STAR), Singapore, Singapore; 2 Department of Psychological Medicine, Yong Loo Lin School of Medicine, National University of Singapore, Singapore, Singapore

## Abstract

Human primary monocytes comprise a heterogeneous population that can be classified into three subsets based on CD14 and CD16 expression: classical (CD14^high^/CD16^−^), intermediate (CD14^high^/CD16^+^), and non-classical (CD14^low^/CD16^+^). The non-classical monocytes are the most pro-inflammatory in response to TLR stimulation *in vitro*, yet they express a remarkably high basal level of miR-146a, a microRNA known to negatively regulate the TLR pathway. This concurrence of a pro-inflammatory status and a high miR-146a level has been associated with cellular senescence in other cell types. Hence, we assessed the three monocyte subsets for evidence of senescence, including proliferative status, telomere length, cellular ROS levels, and mitochondrial membrane potential. Indeed, the non-classical subset exhibited the clearest hallmarks of senescence, followed by the intermediate and then the classical subset. In addition, the non-classical subset secreted pro-inflammatory cytokines basally *in vitro*. The highly pro-inflammatory nature of the non-classical monocytes could be a manifestation of the senescence-associated secretory phenotype (SASP), likely induced by a high basal NF-κB activity and IL-1α production. Finally, we observed an accumulation of the non-classical monocytes, in conjunction with higher levels of plasma TNF-α and IL-8, in the elderly. These factors may contribute to inflamm-aging and age-related inflammatory conditions, such as atherosclerosis and osteoarthritis. With our new understanding that the non-classical monocyte subset is a senescent population, we can now re-examine the role of this subset in disease conditions where this subset expands.

## Introduction

Human primary monocytes comprise a heterogeneous population, of which 10-20% can be distinguished by their expression of surface antigen CD16^1^. These CD16^+^ cells are known as the “inflammatory” subset due to their potent pro-inflammatory activity^2^. *In vitro*, CD16^+^ monocytes produce higher amounts of TNF and minimal IL-10 in response to toll-like receptor (TLR) stimulation, compared to “classical” CD16^−^ monocytes^3–5^. *In vivo*, the CD16^+^ subset expands in various conditions such as asthma, coronary artery diseases and Crohn’s disease, and during infections such as sepsis and hepatitis B^6,7^, signifying the clinical significance of the CD16^+^ subset during inflammation.

Our previous microRNA (miR) profiling study on monocyte subsets found the basal level of miR-146a to be significantly higher in CD16^+^ cells compared to CD16^−^cells^8^. MiR-146a is a negative regulator of the TLR signaling pathway, hence it serves to limit pro-inflammatory responses^9^. The higher miR-146a level in the non-classical CD16^+^ monocytes is not consistent with the pro-inflammatory nature of these cells. These data suggest, therefore, that up-regulation of miR-146a may have other functions other than being a negative regulator of the TLR signaling pathway. Indeed, up-regulated miR-146a has been associated with cellular senescence in various cell types, including human fibroblasts, trabecular meshwork cells and endothelial cells^10–12^.

Cellular senescence is a state of irreversible proliferative arrest^13^. Natural senescence caused by telomere shortening after multiple replications is termed “replicative senescence,” and is synonymous to aging at the cellular level. Cellular senescence can also occur pre-maturely when a cell is exposed to environmental insults (stress-induced senescence), or when an oncogene is activated (oncogene-induced senescence). Although a senescent cell can no longer proliferate, it remains viable and metabolically active. Senescent cells accumulate with age in various species, including rodents, primates, and humans^14,15^. These cells typically undergo extensive changes in protein expression and secretion, resulting in the development of a senescence-associated secretory phenotype (SASP)—the persistent secretion of pro-inflammatory growth factors, cytokines, chemokines, proteases and extra-cellular matrix components^16^. An accumulation of senescent cells exhibiting SASP in tissues thus results in a pro-inflammatory microenvironment.

Immune cells are also known to undergo cellular senescence. A subset of NK cells, the decidual NK cells, undergo senescence upon activation of CD158d by HLA-G, which secreted by fetal trophoblasts during pregnancy^17^. These NK cells then develop a specific SASP that is crucial for promoting immune tolerance and maintaining pregnancy. In T cells, senescence is acquired after prolonged exposure to antigens accumulated with age, or by constant stimulation during a chronic infection, such as cytomegalovirus infection^18^. These T cells lose the expression of CD28, which is a co-stimulatory receptor required for proliferation, and exhibit an SASP which have protective roles against infections. In macrophages, senescence is induced upon activation by pro-inflammatory stimuli, resulting in the polarization to the M1 phenotype^19^. Thus the onset of cellular senescence in different immune cells leads to different cell fates and functions.

The up-regulation of miR-146a, together with the superior pro-inflammatory nature of CD16^+^ monocytes reminiscent of SASP, lead us to speculate that the CD16^+^ monocytes are senescent. This concept may be contrary to what is currently known about primary monocytes. Monocytes are thought to have a short lifespan of about 3 days in the blood and are generally perceived to be non-proliferative during this time^20^. Transcriptomic profiling data, however, have suggested that “classical” CD16^−^ monocytes are proliferative while CD16^+^ monocytes are anti-proliferative^21,22^. Additionally, the existence of a “proliferative monocyte” population has been observed *in vitro*^23^. Importantly, a recent study has shown that human monocytes can circulate in the blood for as long as 12 days^24^. Hence it is credible that monocytes may undergo cellular senescence in the blood during this time.

Here, we investigated the concept that CD16^+^ monocytes are senescent cells. As the CD16^+^ subset consists of CD14^high^ and CD14^low^ subsets, we conducted our study on three monocyte subsets according to the latest classification: classical (CD14^high^/CD16^−^), intermediate (CD14^high^/CD16^+^), and non-classical (CD14^low^/CD16^+^)^25^. We first assessed whether any of these three subsets exhibited features of cellular senescence, including SASP, and then probed the underlying mechanisms of senescence. We also investigated the accumulation of senescent monocytes in elderly subjects, since senescent cells are known to accumulate with age.

## Results

### The high level of miR-146a expression in the non-classical monocytes does not inhibit their response to LPS

**Figure 1:**
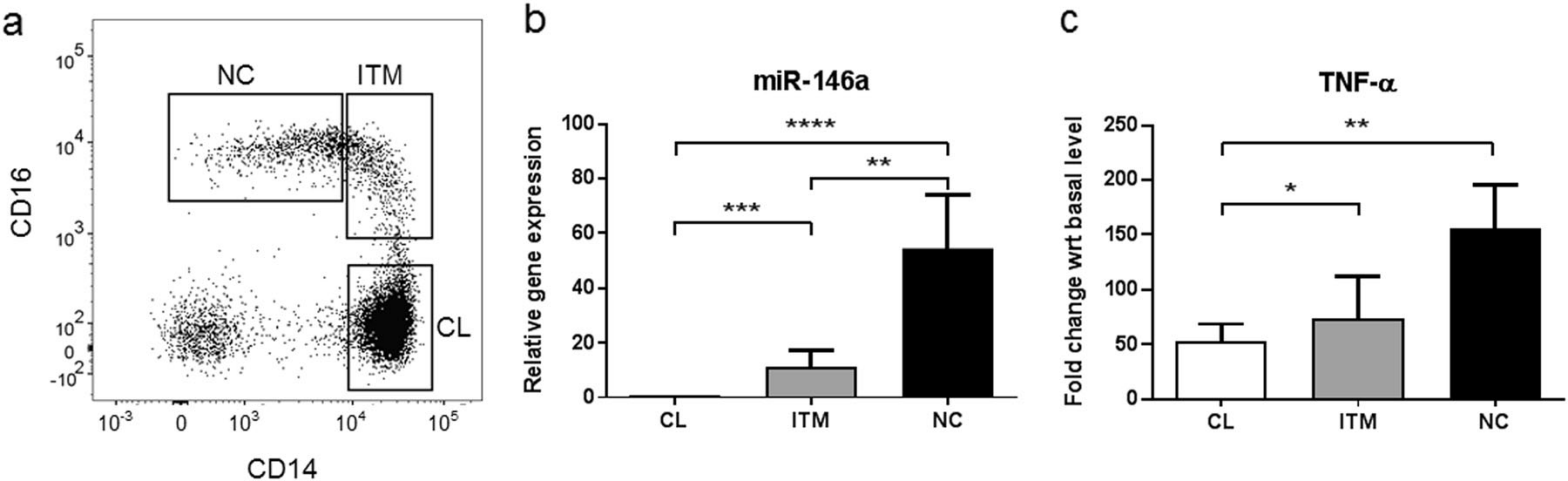
High microRNA-146a (miR-146a) level in non-classical monocytes does not limit the LPS response. **a** Gating of enriched monocytes to generate the three monocyte subsets based on CD14 and CD16 expression. **b** Basal miR-146a expression in the three monocyte subsets. **c** TNF-α mRNA up-regulation in the three monocyte subsets, as an acute response to 2 h LPS stimulation. TNF-α mRNA level expressed as a fold change with respect to basal level before LPS stimulation. The data represent the means ± SD; *n* ≥ 3; *p < 0.05; **p < 0.01; ***p < 0.001; ****p < 0.0001. *CL:* classical, *ITM:* intermediate, *NC:* non-classical

We first enriched for monocytes from peripheral blood mononuclear cells (PBMCs) by depleting the lymphocytes using anti-CD15, anti-CD56, anti-CD3, and anti-CD19-coated microbeads. The enriched monocytes were then sorted by fluorescence-activated cell sorting (FACS) into three subsets: classical (“CL”; CD14^high^/CD16^−^), intermediate (“ITM”; CD14^high^/CD16^+^), and non-classical (“NC”; CD14^low^/CD16^+^) (Fig. 1a). The basal expression of miR-146a was analyzed in each subset. The non-classical subset expressed the highest level of miR-146a, with >3-fold and ~60-fold higher expression than the intermediate and classical subsets, respectively (Fig. 1b). MiR-146a negatively regulates the TLR signaling pathway via post-transcriptional targeting of IRAK1 and TRAF6^9^. To assess the acute pro-inflammatory response to TLR stimulation, we treated the three subsets with LPS for 2 h, and assessed the induction of TNF-α from baseline, at the mRNA level. The non-classical subset showed a 150-fold up-regulation of TNF-α, while the classical and intermediate subsets each showed only ~50-fold up-regulation (Fig. 1c). Hence despite the high level of miR-146a, the non-classical subset is still the most pro-inflammatory subset of the three, producing the most TNF-α in response to LPS stimulation.

### Non-classical monocytes exhibit various features of senescence

The phenomenon that non-classical monocytes produce a high level of miR-146a but remain the most pro-inflammatory monocyte subset suggests an alternative role for miR-146a in these cells. Indeed, miR-146a has been reported to be an independent marker of senescence in various cell types. In conjunction, these senescent cells exhibit a pro-inflammatory behavior due to SASP^10–12^. We thus examined whether non-classical monocytes represent a senescent monocyte subset.

**Figure 2:**
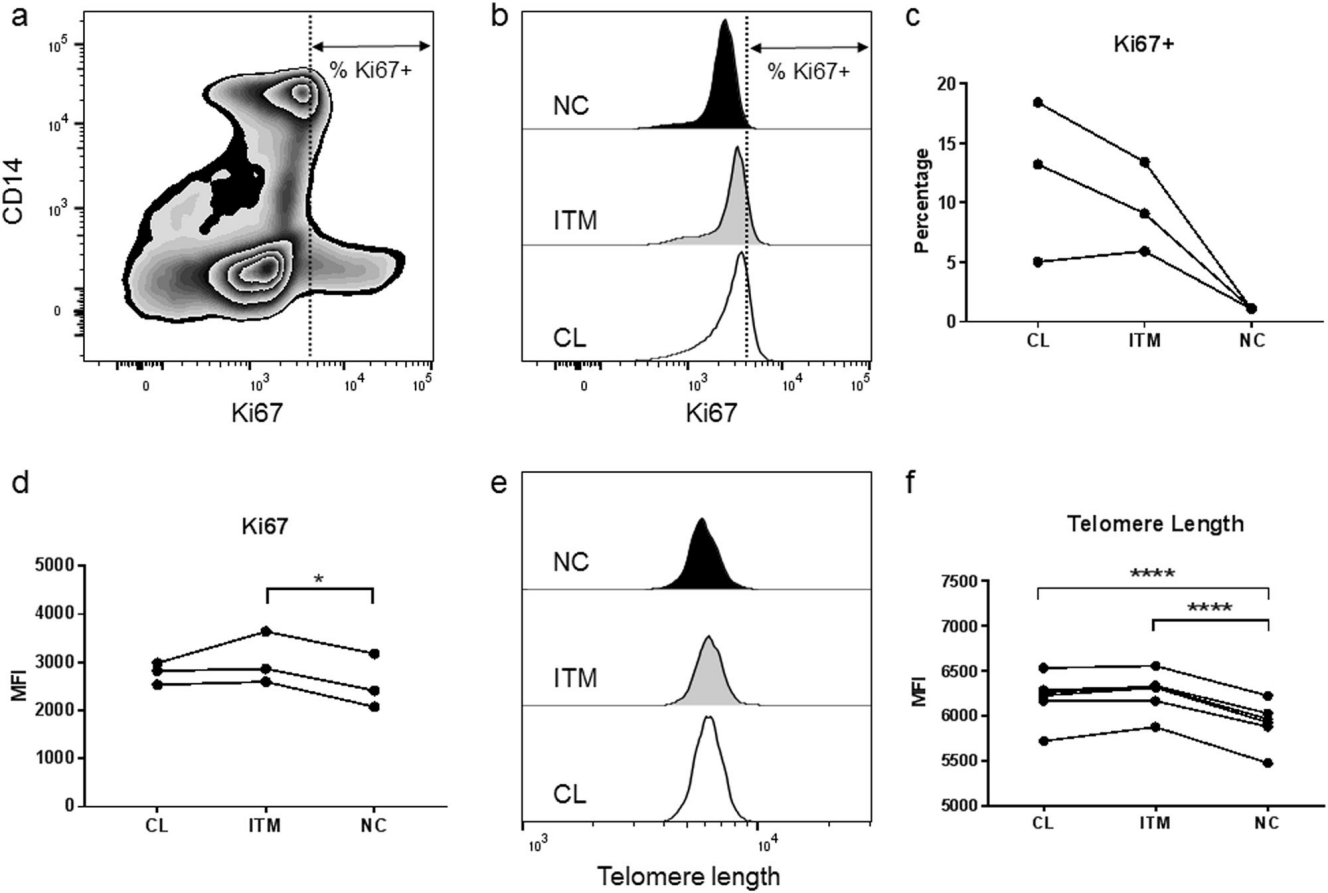
Non-classical monocytes are the least proliferative. **a** Expression of Ki67 in total peripheral blood mononuclear cells. Ki67^+^ cells were gated using actively proliferating lymphocytes as a guide. **b**–**c** Percentage of Ki67^+^ cells in the three monocyte subsets. **d** Relative expression levels of Ki67 in the three monocyte subsets. **e**-**f** Relative telomere length in the three monocyte subsets. Each line represents one donor; *n* ≥ 3. *p < 0.05; ****p < 0.0001. *CL*: classical, *ITM:* intermediate, *NC:* non-classical, *MFI:* median fluorescence intensity

We first investigated Ki67 expression and telomere length in the three monocyte subsets. Ki67 is a protein that is exclusively expressed by proliferating cells, while telomere length of chromosomes shortens with every proliferative cycle. Using PBMCs, we identified the subsets with the gating strategy described in Supplementary Figure S1. The non-classical subset had the lowest percentage of Ki67^+^ cells (Figs. 2a-c) and the lowest level of Ki67 expression (Fig. 2d), as well as the shortest telomere length (Figs. 2e, f). These features suggested that the non-classical subset is the least proliferative and most senescent subset.

To confirm our findings, we investigated additional features of senescence. As a cell progresses towards senescence, cellular ROS levels increase, resulting in electron transport chain dysfunction and a drop in mitochondrial membrane potential (MMP)^26^. In addition, high levels of cellular ROS can sustain the activation of ERK, which is reportedly observed in senescent cells^27^. Hence we assessed the cellular ROS levels, MMP, and phosphorylated ERK (p-ERK) levels in the three monocytes subsets.

**Figure 3:**
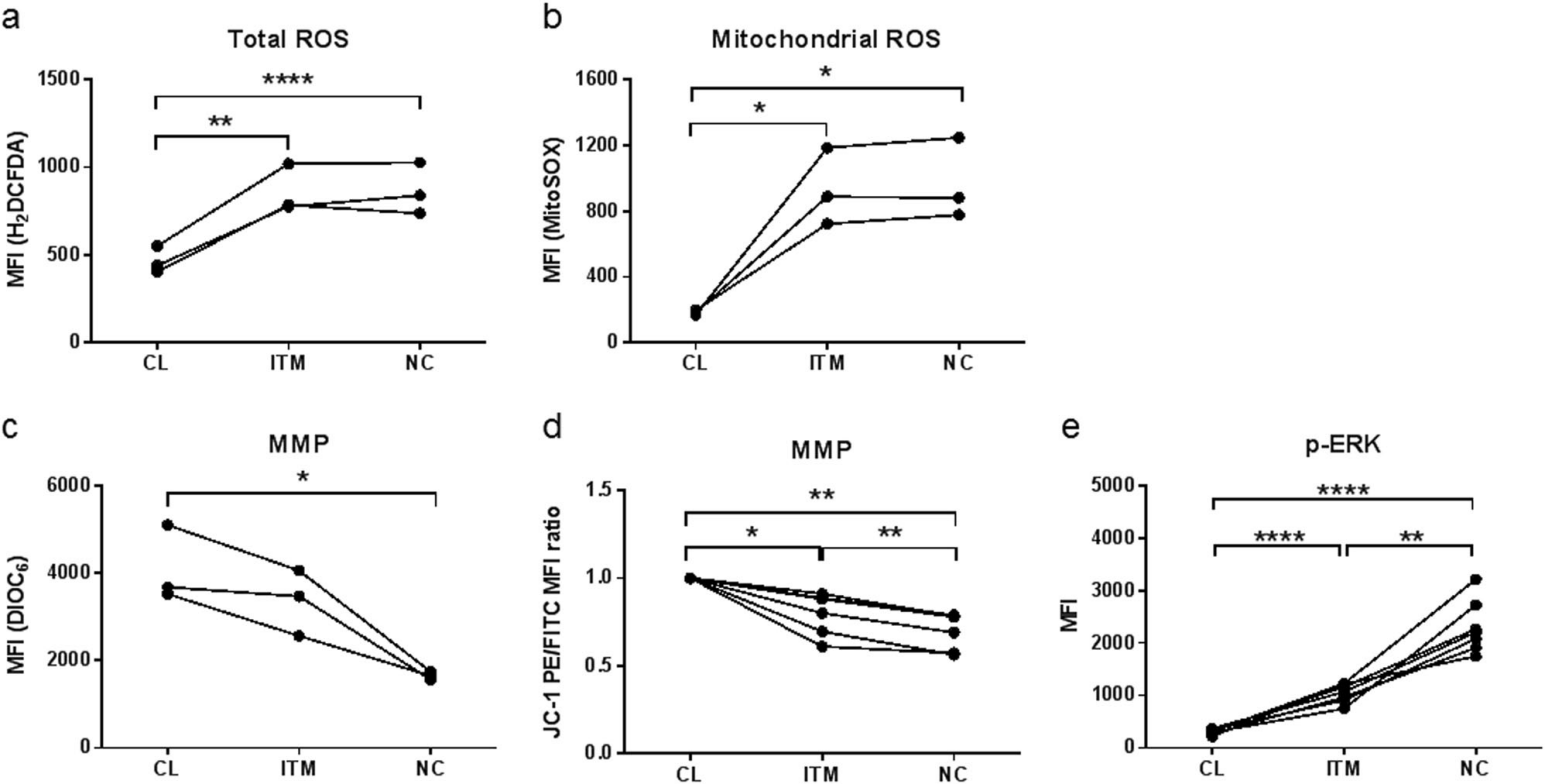
Non-classical monocytes exhibit several features of senescence. **a** Relative total cellular ROS levels as measured using H_2_DCFDA ROS indicator. **b** Relative mitochondrial ROS levels as measured using MitoSOX mitochondrial superoxide indicator. **c**–**d** Relative mitochondrial membrane potential (MMP) as measured using DIOC_6_ and JC-1. **e** Relative expression levels of p-ERK. All the parameters were measured using flow cytometry. Each line represents one donor; *n* ≥ 3. *p < 0.05; **p < 0.01; ****p < 0.0001. *CL:* classical, *ITM:* intermediate, *NC*: non-classical, *MFI:* median fluorescence intensity, *MMP*: mitochondrial membrane potential, *ROS*: reactive oxygen species

The non-classical subset had a high level of total cellular ROS, which was similar to that of the intermediate subset but double that of the classical subset (Fig. 3a). A similar trend was observed for mitochondrial ROS (Fig. 3b). In line with the high ROS levels, the non-classical subset exhibited the lowest MMP, as measured by both DIOC_6_ and JC-1 compounds, which was followed by the intermediate subset, and then the classical subset (Figs. 3c, d). Finally, p-ERK levels in the non-classical subset were >3 times the level of the other two subsets (Fig. 3e). Together, these features indicate that non-classical monocytes comprise the most senescent subset, followed sequentially by the intermediate and then the classical subset.

### Non-classical monocytes exhibit SASP *in vitro* and *in vivo*

**Figure 4:**
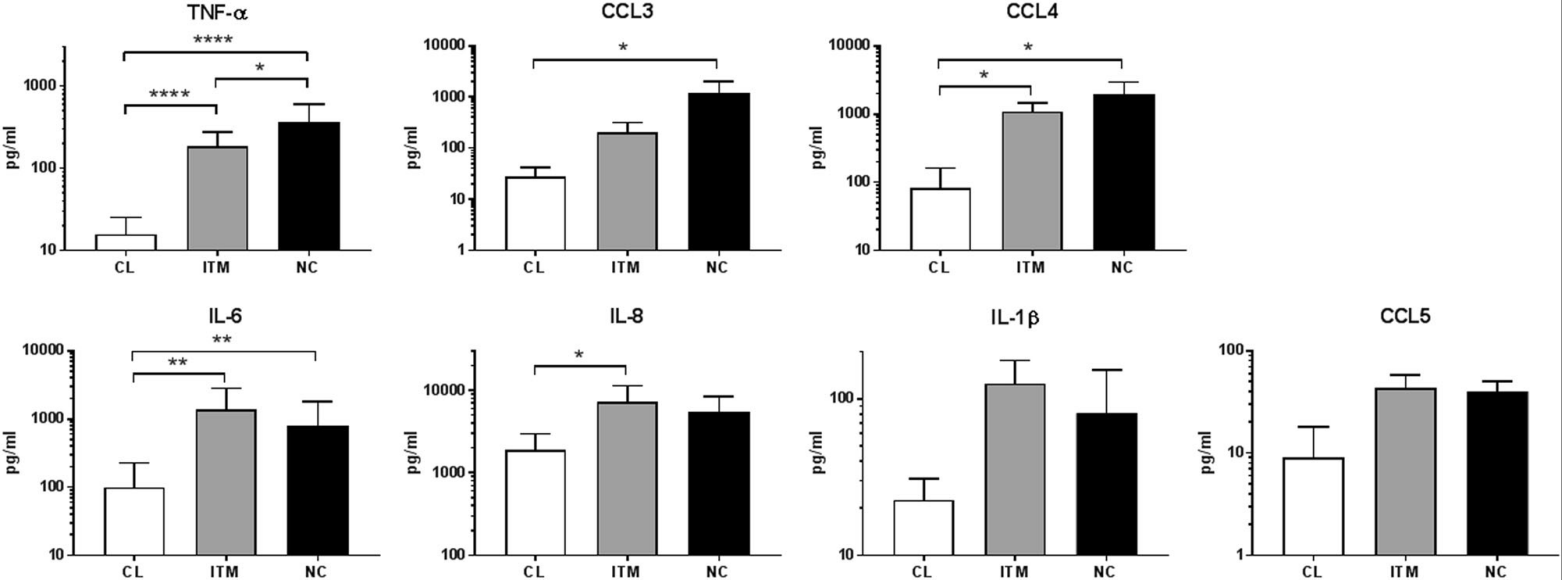
Non-classical and intermediate monocytes secrete high levels of pro-inflammatory cytokines basally. TNF-α, CCL3, CCL4, CCL5, IL-6, IL-8 and IL-1β secretion was analyzed by Luminex assay after overnight culture. The data represent the means ± SD; *n* = 3. *p < 0.05; **p < 0.01; ****p < 0.0001. *CL:* classical, *ITM:* intermediate, *NC:* non-classical

**Figure 5:**
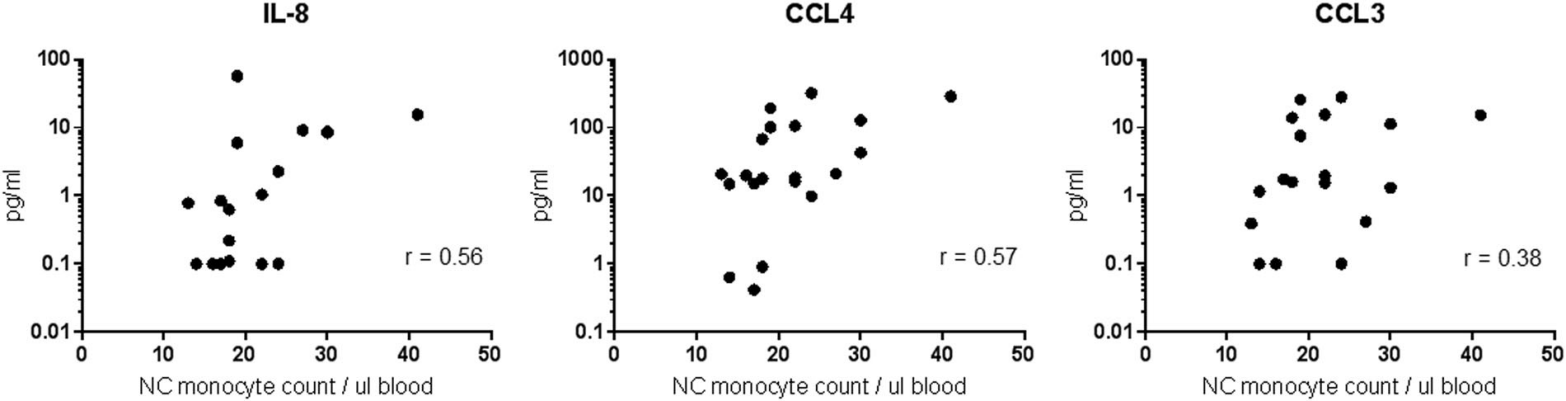
Plasma levels of cytokines correlate with non-classical monocyte count in the blood. IL-8, CCL4 and CCL3 levels in the plasma was analyzed by Luminex assay, and correlated with the absolute number of non-classical monocytes present in a µL of whole blood. Each dot represents one donor; *n* = 20. *NC:* non-classical

SASP is the secretion of pro-inflammatory factors into the microenvironment by a senescent cell. These factors include growth factors, cytokines, chemokines, proteases and extra-cellular matrix components^16^. We therefore assessed the basal cytokine secretion profile of the three monocyte subsets. Monocytes were sorted (by FACS) into three subsets and cultured overnight. The non-classical subset secreted the highest levels of TNF-α, CCL3, CCL4, while IL-6, IL-8, IL-1β and CCL5 were secreted at equally high levels in both the intermediate and non-classical subsets (Fig. 4). Notably, the classical subset secreted low levels of all these cytokines compared to the two CD16^+^ subsets. To see the effects of SASP of monocytes *in vivo*, we screened 20 healthy volunteers for the levels of SASP cytokines in their plasma and correlated them with the count of non-classical monocytes in the blood. We found that three cytokines, IL-8, CCL4, and CCL3, showed a positive correlation with the number of non-classical monocytes in the blood (Fig. 5). Together, these data suggest that the two CD16^+^ subsets exhibit a pro-inflammatory secretory phenotype reminiscent of SASP, and the presence of more non-classical monocytes may contribute to an increase in blood plasma levels of cytokines.

### NF-κB and membrane-bound IL-1α is abundant on non-classical monocytes

**Figure 6:**
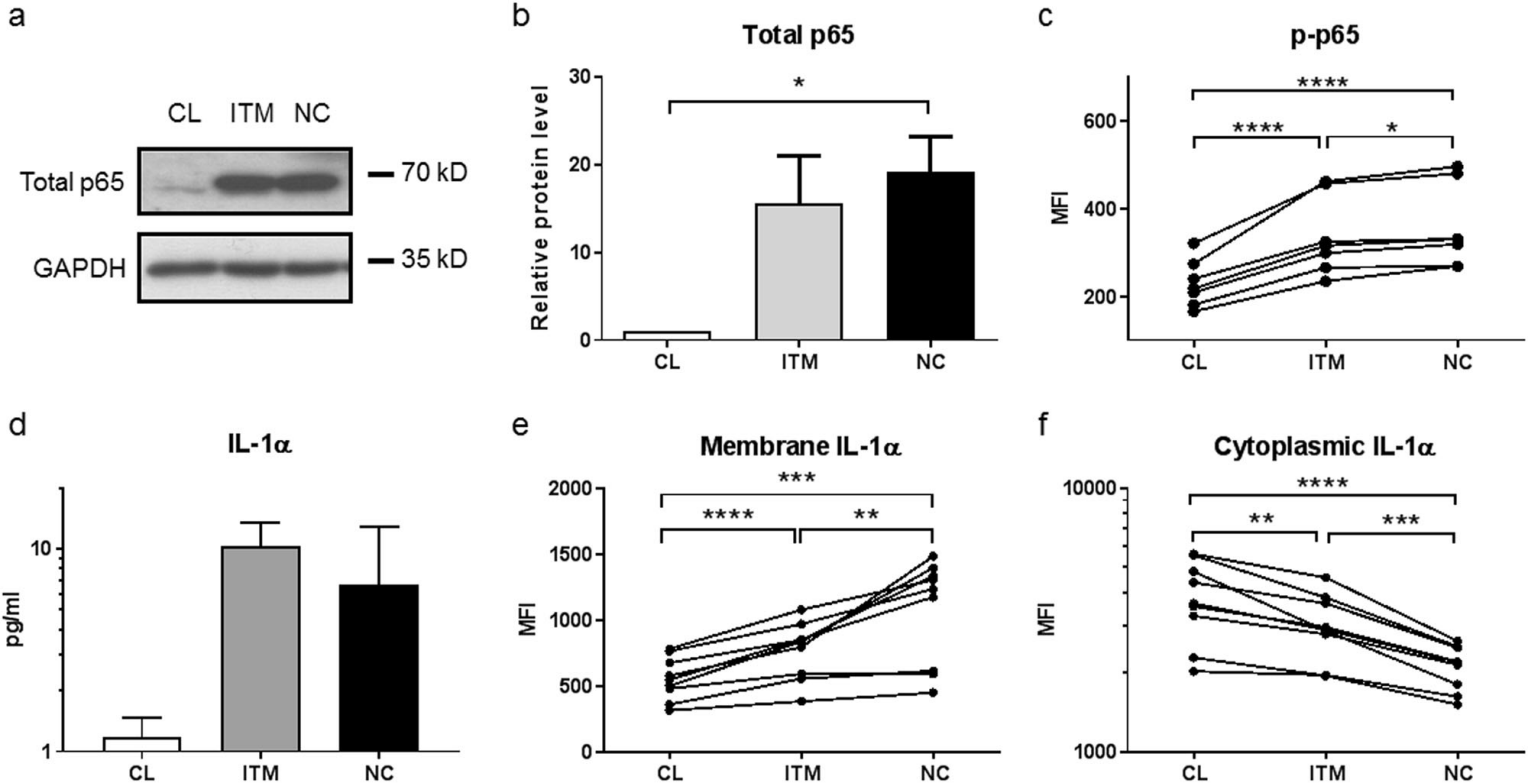
Non-classical and intermediate monocytes express high levels of NF-κB (p65) and membrane-bound IL-1α. **a** Western blot analysis of total p65 and GAPDH protein levels in the three monocyte subsets. **b** Quantification of Western blot data shown in (**a**): p65 protein level was normalized to GAPDH (loading control) and expressed as a fold change with respect to CL subset. The data represent the means ± SD; *n* = 3. **c** Relative levels of phosphorylated-p65 (p-p65), measured by flow cytometry. Each line represents one donor; *n* = 7. **d** IL-1α secretion by the three monocyte subsets, measured by Luminex assay. The data represent the means ± SD; *n* = 3. **e**–**f** Relative expression levels of membrane-bound IL-1α (**e**) and cytoplasmic IL-1α (**f**), analyzed by flow cytometry. Each line represents one donor; *n* = 9. *p < 0.05; **p < 0.01; ***p < 0.001; ****p < 0.0001. *CL:* classical, *ITM:* intermediate, *NC:* non-classical, *MFI:* median fluorescence intensity

We next investigated the mechanistic pathway leading to SASP in monocytes. As NF-κB is a transcription factor for many pro-inflammatory cytokines and the main inducer of SASP^28^, we assessed the basal activation level of NF-κB (p65) in the three monocyte subsets. Indeed, the non-classical subset expressed the highest levels of both total (Figs. 6a, b) and, more importantly, phosphorylated p65 (p-p65) compared to the other two subsets (Fig. 6c).

IL-1α is reported to be the upstream regulator of NF-κB, which induces SASP in human fibroblasts. But instead of being secreted, IL-1α is bound to the cell membrane of senescent human fibroblasts^29^. We thus explored IL-1α as a possible SASP inducer in the monocytes. Indeed, secretion of IL-1α by all three monocyte subsets was minimal (Fig. 6d). Instead, membrane-bound IL-1α was detected on all three monocyte subsets, with the highest level found on the non-classical subset, followed by the intermediate and then the classical subset (Fig. 6e). Interestingly, the cytoplasmic levels of IL-1α were opposite to the membrane levels of IL-1α, with the non-classical subset exhibiting the lowest level cytoplasmic IL-1α of the three subsets (Fig. 6f), suggesting that the majority of IL-1α produced by the non-classical subset has been preferentially transported to the cell membrane. Together, these results indicate that the IL-1α–SASP pathway is active in the non-classical subset.

### Exogenous IL-1α can induce SASP in classical monocytes

**Figure 7:**
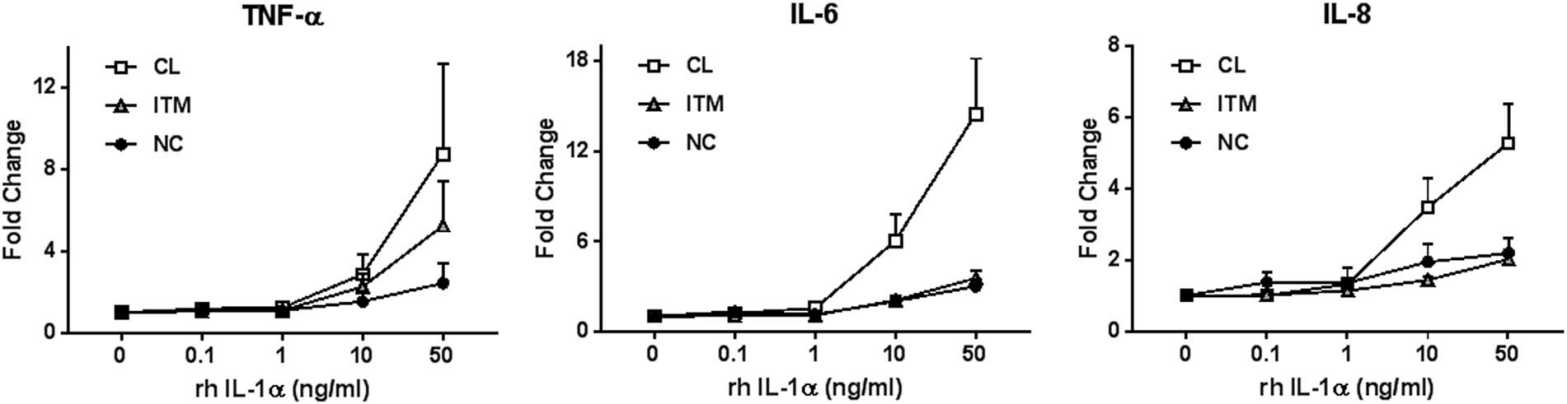
Classical monocytes can be induced to exhibit senescence-associated secretory phenotype following exposure to rh IL-1α. Fold change increase, with respect to no treatment, in TNF-α, IL-6, and IL-8 secretion in the three monocyte subsets. The data represent the means ± SD; *n* = 3. *CL:* classical, *ITM:* intermediate, *NC:* non-classical, *rh*: recombinant human

We next investigated if treatment with IL-1α could induce SASP in the classical monocytes. Indeed, we found that recombinant human (rh) IL-1α treatment induced a robust dose-dependent increase in the production of SASP cytokines, mainly TNF-α, IL-6, and IL-8 in the classical subset (Fig. 7). The intermediate and non-classical subsets showed only a modest response to the IL-1α treatment. As these two subsets already exhibit SASP, we speculate that the pathway is saturated and thus cannot be further induced.

We also wanted to determine whether we could inhibit the SASP of the non-classical monocytes by interfering with the IL-1α pathway. To this aim, we used an rh IL-1 receptor antagonist (IL-1Ra) or an anti-IL-1α neutralizing antibody. IL-1Ra binds IL-1R, making the receptor unavailable for binding IL-1α, without activating the IL-1α pathway, whereas the anti-IL-1α antibody sequesters unbound IL-1α and prevents it from binding to the receptor. Unfortunately, treatment with either rh IL-1Ra or anti-IL-1α had no effect in reducing SASP in the non-classical subset (Supp Fig. S2 and S3). These data suggests that once the IL-1α pathway has been activated, it cannot be easily inhibited.

### Senescent, non-classical monocytes accumulate in the elderly

**Figure 8:**
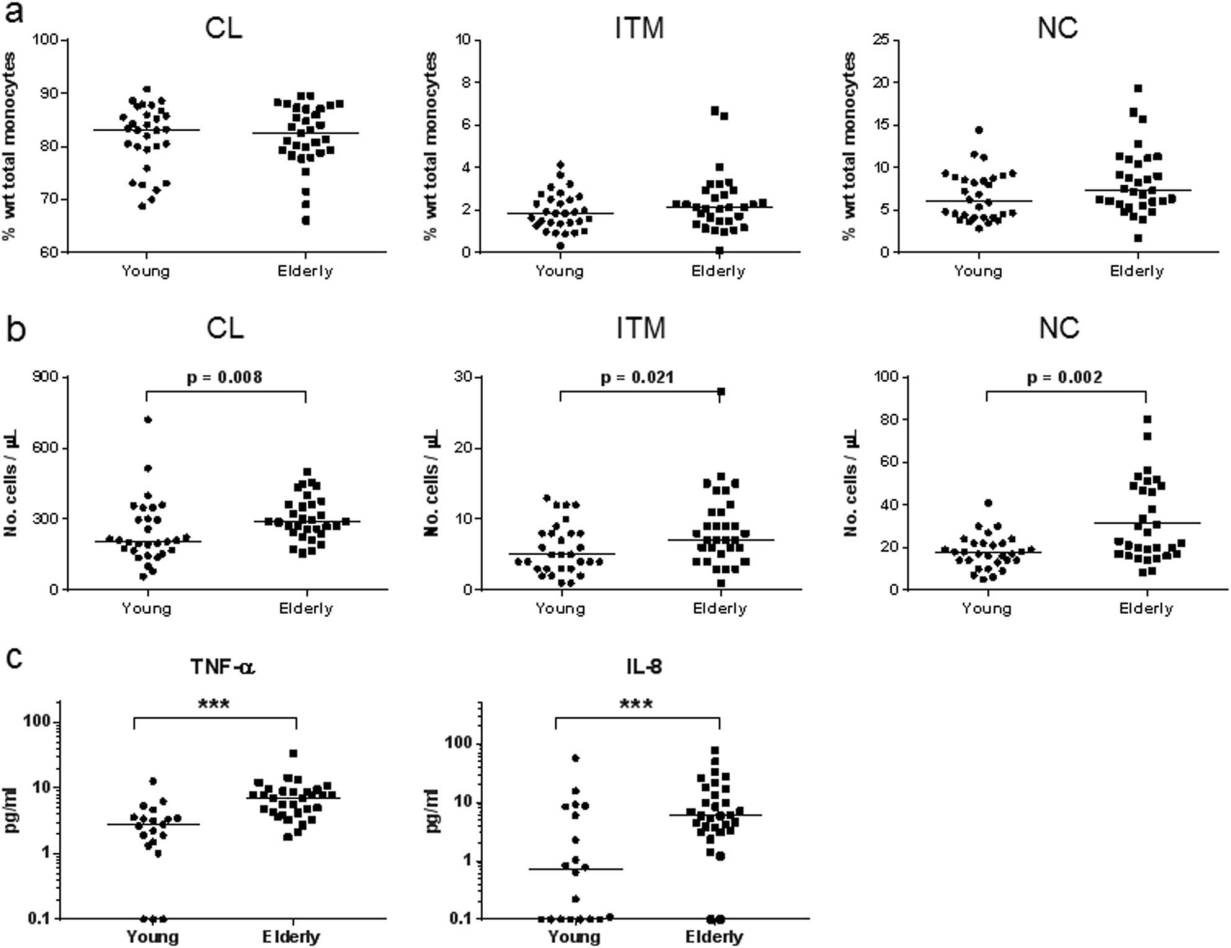
The elderly has higher non-classical monocyte count and plasma level of SASP cytokines. **a** Number of monocytes from each subset expressed as a percentage of the total number of monocytes. **b** Absolute number of monocytes from each subset present in a µL of whole blood. **c** TNF-α and IL-8 levels in the plasma, analyzed by Luminex assay. Each dot represents one donor; line represents median; (**a**–**b**) *n* = 30 each for both young and elderly individuals; (**c**) *n* = 20 for young, *n* = 30 for elderly; ***p < 0.001. *CL:* classical, *ITM*: intermediate, *NC:* non-classical

Senescent cells accumulate with age^15^, and are thought to contribute to “inflamm-aging”—a chronic, low-level systemic inflammation observed in the elderly^30^. We thus hypothesized that the non-classical subset accumulates in the elderly. We recruited 30 healthy, young volunteers aged 22–35 years, and 30 healthy, elderly volunteers aged > 55 years old. In terms of percentage of total monocytes, we found no significant difference in all three subsets between the young and the elderly cohorts (Fig. 8a). However, in terms of monocyte count per volume blood, the elderly showed an increase in all three subsets, especially in the non-classical subset (*p* = 0.002) (Fig. 8b). We next investigated if the accumulation of non-classical monocytes in the elderly would lead to a higher level of SASP cytokines in the plasma. Indeed, the level of cytokines in the plasma of the elderly was generally higher (Supp Fig. S4), with the levels of TNF-α and IL-8 being significantly higher (Fig. 8c). These data show that senescent monocytes indeed accumulate in the elderly and could contribute to changes in the blood cytokine levels.

## Discussion

MiR-146a is a principal negative regulator of the TLR signalling pathway. In monocytic cell lines, up-regulation of miR-146a alone is sufficient to inhibit the response to LPS stimulation, while knockdown alone can restore the response^31^. However, we saw that the high miR-146a level in the non-classical monocytes, compared to the intermediate and classical monocytes, did not inhibit their response to LPS (Fig. 1); instead, the non-classical monocytes exhibited the greatest response to LPS. This indicated that the high basal expression of miR-146a in non-classical monocytes may have other functions. Indeed, numerous studies have shown that miR-146a is a marker of cellular senescence in various cell types^10–12^.

Cellular senescence is a state of irreversible proliferative arrest in response to diverse stresses^13^. We found that the non-classical monocytes are the least proliferative subset, expressing the lowest level of Ki67 (Fig. 2), supporting the notion that non-classical monocytes are senescent. We also saw that the non-classical monocytes have the shortest telomere length, indicating that they have undergone more replications than the other two subsets. Although monocytes are generally perceived to be non-proliferative cells, transcriptomic profiling data suggested that classical monocytes are proliferative and anti-apoptotic, compared to the intermediate and non-classical subsets^21,22^. Additionally, the existence of a “proliferative monocyte” population *in vitro* was reported, and this population was identified as CD16^−^ classical monocytes^23^. As the concept of a senescent subset of monocytes is rather novel, we used a combination of markers to comprehensively profile the cells, namely ROS levels, mitochondrial membrane potential and p-ERK levels (Figs. 2, 3). Altogether, these markers indicated that there was a progressive transition towards senescence from the classical to the intermediate to the non-classical subset. Moreover, the non-classical and intermediate subsets exhibited a pro-inflammatory secretory profile that was indicative of SASP (Fig. 4), further reinforcing their senescence status. The SASP of non-classical monocytes is likely induced by high basal levels of phosphorylated NF-κB (p65) (Fig. 6), which is a transcription factor for pro-inflammatory cytokines, and incidentally, also miR-146a. In monocytic cell lines and fibroblasts, p65 up-regulates miR-146a concomitantly with pro-inflammatory cytokine production, in a negative feedback mechanism to limit excessive inflammation^9,10^. The high p-p65 levels in the non-classical and intermediate subsets may likely underlie their pro-inflammatory secretory profile and high miR-146a levels.

Cellular senescence is also considered a characteristic of terminally-differentiated cells, as in the case of decidual NK cells in pregnancy, late-differentiated T cells after repeated stimulation^32^, and fibroblasts after inflammation^33,34^. Recently, post-mitotic terminally-differentiated cells such as neurons and adipocytes are also found to exhibit features of senescence^35^. Similarly, the non-classical monocytes may be at an advanced stage of differentiation. In fact, for the last two decades, the CD16^+^ monocytes have been speculated to be more differentiated than their CD16^−^ counterparts, based on their phenotypic marker expression^36,37^, functional characteristics^3,22,38^, and transcriptomic profiles^21,36^. Recently, a developmental relationship between the three monocyte subsets was elegantly shown using *in vivo* deuterium labeling of monocytes; restoration of blood monocytes following monocytopenia (a state of reduced monocytes in the blood) began with the appearance of classical monocytes first, followed by intermediate monocytes, and lastly non-classical monocytes^24^, indicating that monocytes emerge from the bone marrow as classical monocytes, and with time differentiate into intermediate and then non-classical monocytes. Hence the senescent status of the non-classical monocytes may symbolize blood monocytes that are terminally-differentiated.

The accumulation of senescent cells with age in various tissues such as skin, chondrocyte clusters and arterial endothelium, in both human and animals, is well-described in the literature^14,15,39,40^. In the human immune system, accumulation of senescent T cells, B cells, and even hematopoietic stem cells with age has been reported^32^. However, for monocytes, contradicting reports exist for the accumulation of any subset with age^41–44^, likely due to technical differences between studies, such as sample processing techniques and gating strategies used for flow cytometry. For this reason, we minimized sample processing by staining for monocyte subsets in whole blood samples. While we saw an overall increase in the number of monocytes in all three subsets in the elderly, the most significant increase occurs in the non-classical subset (Fig. 8b), consistent with the concept that senescent cells accumulate with age. It is interesting to speculate the underlying mechanisms for the increase in the number of monocytes in the elderly, and particularly, the non-classical monocytes. We propose two possibilities: Firstly, there could be a delayed clearance of non-classical monocytes due to an altered apoptotic program. Secondly, there could be an accelerated senescence of monocytes induced by inflamm-aging, which is a low-level systemic inflammation normally present in the elderly^30^. An accumulation of non-classical monocytes in the elderly can in turn contribute to inflamm-aging, as we saw that the number of non-classical monocytes positively correlates with level of pro-inflammatory cytokines in the blood (Fig. 5). Inflamm-aging is postulated to play an important role in the progression of age-related degenerative conditions such as atherosclerosis, osteoarthritis, sarcopenia, type two diabetes mellitus and even cancer^42,45,46^.

Other than in the elderly, an accumulation of the CD16^+^ monocytes has also been reported for various pathologies resulting from chronic inflammation, such as systemic sclerosis^47^, liver fibrosis^48^, kidney disease^49^, atherosclerosis^50^, and psoriatic arthritis^51^. Notably, these senescent monocytes appear to aggravate the conditions, possibly due to their SASP. In systemic sclerosis, an increase in the number of CD16^+^ monocytes was associated with fibrotic manifestations of the disease^47^; and in liver injury, the number of CD16^+^ monocytes positively correlates with the extent of liver fibrosis^48^. In atherosclerosis, senescent foam cell macrophages—likely derived from CD16^+^ monocytes^52,53^—are shown to drive atheroma formation and pathology^54^. Hence, accumulation of senescent monocytes is implicated in various clinical conditions. Therefore, we may propose that reducing SASP from non-classical monocytes would reduce the chronic inflammatory status and hence be a possible therapeutic option for these conditions. Unfortunately, blocking IL-1α was insufficient to reduce SASP in the non-classical monocytes (Supp Fig. S2 and S3) although we found that SASP in the monocytes was induced by IL-1α (Fig. 7). Thus an alternative strategy would be eliminating the source of SASP—the non-classical monocytes. This idea derives from studies using progeroid mouse models (mice with accelerated physiological aging due to genetic disorders), where elimination of senescent cells delayed the presentation of age-related disorders^55^. Similarly, in humans, we may be able to reduce systemic inflammation by reducing the number of circulating non-classical monocytes. Currently in the clinic, there are already three therapies, namely the glucocorticoid therapy, the intravenous immunoglobulins therapy and the MCSF pathway blockade therapy, that have been shown to reduce the number of non-classical monocytes in patients^56–58^. Glucocorticoid therapy has been used as an immunosuppressive and anti-inflammatory agent for treating multiple sclerosis and atherosclerosis; intravenous immunoglobulins therapy has been in use for treating common variable immunodeficiency patients; and MCSF pathway blockade therapy using human monoclonal antibodies against MCSF was in clinical trials for treating rheumatoid arthritis. These therapies can potentially benefit the elderly and patients with other chronic inflammatory conditions related to expansion of the non-classical subset.

In conclusion, non-classical monocytes represent a subset of senescent monocytes in our circulation and may be a novel potential target for therapy in age-related and chronic inflammatory conditions.

## Materials and methods

### Patient consent and ethical review

Human blood sample collection and all experimental procedures were approved by the Institutional Review Board, Singapore, in accordance with the guidelines provided by the Health Sciences Authority of Singapore. Informed written consent was obtained from participants for this study in accordance with the Declaration of Helsinki. Elderly study participants were recruited from the Singapore Longitudinal Aging Study Wave 2, which is an ongoing population-based cohort study of aging and health among Chinese elderly adults aged 55 years and above^42^. Participants aged 22–35 years were enrolled from the Singapore Immunology Network and constituted the “young” cohort.

### Purification of blood monocyte subsets

PBMCs were obtained by Ficoll density gradient centrifugation and were then depleted of granulocytes and lymphocytes using anti-CD15, anti-CD56, anti-CD3, and anti-CD19 microbeads (Miltenyi Biotec). The enriched monocyte fraction was then labeled with anti-CD14 [61D3] (#48-0149-42; eBioscience), anti-CD16 [VEP13] (#130-098-099; Miltenyi Biotec) and anti-CD56 [NCAM16.2] (#340363; BD Biosciences), for sorting by fluorescence-activated cell sorting (FACS) into the three monocyte subsets. For FACS, we first gated on single cells and then gated on the enriched monocytes population. We then excluded the CD14^−^CD56^+^ NK cells, as they also express CD16 (Supp Fig. S1). The remaining cells were gated into classical (CL; CD14^high^/CD16^−^), intermediate (ITM; CD14^high^/CD16^+^) and non-classical (NC; CD14^low^/CD16^+^) subsets (Fig. 1a).

### Cell culture

Monocytes were cultured in Iscove’s Modified Dulbecco’s Medium (Hyclone) supplemented with 5% human serum (Innovative Research) and 1% penicillin/streptomycin (Invitrogen). Where lipopolysaccharide (LPS) stimulation was performed, 10 ng/ml LPS (*E. coli* serotype O111:B4) was added as indicated.

### Quantitative real-time PCR (qPCR)

Total RNA was isolated from individual subsets (sorted by FACS) using miRCURY RNA Isolation Kit (Exiqon). For micro-RNA, reverse transcription was performed using Universal cDNA Synthesis Kit II (Exiqon), and miR-146a expression was determined by real-time qPCR using ExiLENT SYBR® Green (Exiqon) on ABI7900 apparatus (Applied Biosystems). MiR-146a expression was analyzed in triplicate and normalized to the small-nucleolar RNA 48 (*RNU48*) housekeeping microRNA. Primers for both miR-146a and RNU48 are obtained from Exiqon.

For mRNA expression, reverse transcription was performed using iScript Reverse Transcription Supermix (Bio Rad). TNF-α expression was determined by real-time qPCR using KAPA SYBR FAST ABI Prism (Kapa Biosystems). Expression of TNF-α was analyzed in triplicate on ABI7900 apparatus (Applied Biosystems) and normalized to hypoxanthine-guanine phosphoribosyltransferase (*HPRT*) housekeeping gene. Primer sequences: *TNF-α* fw: CTG CAC TTT GGA GTG ATC GG; *TNF-α* rv: GGG TTT GCT ACA ACA TGG GC; *HPRT* fw: CTT TGC TTT CCT TGG TCA GG; *HPRT* rv: CAA GGG CAT ATC CTA CAA CAA AC.

### Flow cytometry assays

PBMCs were used for the following flow-cytometric analyses. The three monocyte subsets were identified using the gating strategy shown in Supplementary Figure S1. Samples were analyzed on a BD LSR II flow cytometer (BD Biosciences) and the data were analyzed using FlowJo software (TreeStar).

#### Telomere length measurements

PBMCs were pre-stained with CD14-QD800 [TUK4] (#Q10064; Invitrogen) and CD16-FITC [VEP13] (#130-098-099; Miltenyi Biotec) antibodies. The fluorochromes and clones used to pre-stain the PBMCs were first tested for their ability to withstand the harsh steps in the flow-fluorescent in-situ hybridization (FISH) procedure. For telomere length measurement by FISH, cells were fixed in 1 mM BS3 (Thermo Scientific UK) for 30 min on ice, and quenched with 50 mM Tris in PBS (pH 7.2) for 20 min at room temperature. The cells were then washed in PBS, and then in hybridization buffer [70% deionized formamide, 28.5 mM Tris HCL (pH 7), 1.4% BSA and 0.2 M NaCl], and re-suspended in hybridization buffer containing 0.75 μg/ml PNA TelC-Cy5 FISH probe (Panagene) and incubated at 82 °C for 10 min. The cells were then rapidly cooled on ice and left to hybridize for 1 h at room temperature in the dark before being washed twice in post hybridization buffer [70% deionized formamide, 14.25 mM Tris HCL (pH 7), 0.14% BSA, 0.2 M NaCl, 0.14% Tween20] and twice in 2% BSA in PBS.

For all the following measurements, PBMCs were pre-stained with CD14 [61D3], CD16 [VEP13] and CD56 [NCAM16.3] antibodies for 30 min at room temperature.

#### Ki67 staining

Cells were fixed with the Foxp3/Transcription Factor Staining Buffer Set (eBioscience) and then incubated with anti-Ki67 [Ki67] antibody (#350510; Biolegend) for 30 min at room temperature.

#### MMP measurement

Cells were incubated with 10 nM DiOC6 (Enzo) or 5 μg/ml JC-1 dye (Invitrogen) in culture medium for 30 min at 37 °C, and then washed with PBS. For JC-1 staining, the ratio of the mean fluorescence intensity for the phycoerythrin channel to the FITC channel was calculated to correct for variations in the level of JC-1 uptake and cell size. This ratio was expressed as a fold change with respect to CL subset.

#### Cellular ROS measurement

The cells were incubated with 2.5 μM H_2_DCFDA (Invitrogen) or 5 μM MitoSOX (Invitrogen) in culture medium for 30 min at 37 °C, and then washed with PBS.

#### Membrane IL-1α assessment

The cells were stained with anti-IL-1α (membrane form) [3405] (#FAB200F; R&D Systems) together with the pre-stain antibodies described above. For cytoplasmic IL-1α, pre-stained cells were fixed using the Cytofix/Cytoperm kit (BD Biosciences) for 20 min at 4 °C, before staining with anti-IL-1α (cytoplasmic forms) [4414] (#IC200F; R&D Systems) in Perm/Wash Buffer (BD Biosciences) for 30 min at room temperature.

#### Phosphorylated ERK and p65

Whole blood was fixed with 16% formaldehyde (Thermo Scientific) for 10 min at room temperature. Pre-warmed lysis and permeabilization buffer (0.114% Triton X-100 in PBS) was then added and the samples incubated for 15 min at 37 °C. The cells were then washed in cold wash buffer (4% FBS in PBS), and re-suspended in ice-cold 50% MetOH in PBS for 10 min at 4 °C. The cells were then washed with cold wash buffer, and then stained with anti-CD14 [RMO52], (#A22331, IOTest), anti-CD16 [B73.1] (#12-0167-42, eBioscience), CD45 [HI30] (#45-0459-42, eBioscience) and anti-p-ERK1/2 (pT202/pY204) [20A] (#562644; BD Biosciences) or anti-p-p65 (p-S529) [K10-895.12.50] (#558422; BD Biosciences) in wash buffer for 30 min at room temperature.

#### Elderly versus young study design

Whole blood (100 µl) was stained with CD45 (#25-0459-72, eBioScience), CD14, CD16, CD56 in BD Trucount Absolute Counting Tubes (BD Biosciences) for 15 min at room temperature. 900 µl 1 × BD FACS Lysing solution (BD Biosciences) was then added to the tube and incubated for 15 min at room temperature before analyzing on the flow cytometer.

### Luminex assay

Monocyte subsets (sorted by FACS) were cultured overnight without any stimulation and the cell culture supernatant was collected and frozen in −80 °C until ready for assay. A customized human 20-plex kit (Merck Millipore) was used, consisting of the following targets: IL-1α, IL-6, IL-8, TNF-α, CCL2, CCL3, CCL4, CCL5, CXCL1, IL-1Ra, VEGF, CX3CL1, IL-1β, IL-10, IFN-α, G-CSF, GM-CSF, IFN-γ, EGF, FGF. The assay was performed according to the manufacturer’s protocol. The samples and standards were added to DropArray-bead plates (Curiox) and incubated at 4 °C overnight with fluorescent-coded magnetic beads pre-coated with capture antibodies. The plates were then washed with wash buffer (provided in kit), and the complex was incubated with biotinylated detection antibodies for 1 h at room temperature and then a further incubation with Streptavidin-PE for 30 min at room temperature. The plates were then re-washed and the beads were re-suspended with sheath fluid (Millipore) in PCR plates before reading on the FLEXMAP® 3D Luminex analyzer (Merck Millipore). The data was acquired using xPONENT® 4.0 software (Luminex) and analyzed with Bio-Plex Manager® 6.1.1 software (Bio-Rad).

### Western blot

Cells from monocyte subsets (sorted by FACS) were lysed in RIPA buffer (Sigma) containing 1 × protease inhibitors (Roche). Lysates were loaded onto 10% SDS-polyacrylamide gels (3 × 10^5^ cells per lane). After transfer, the membranes were probed with anti-RelA, dilution 1:200, [532301] (#MAB5078; R&D Systems) or anti-GAPDH, dilution 1:2000, [FF26A/F9] (#649201; Biolegend), followed by HRP-conjugated anti-mouse secondary antibody, dilution 1:5000, (#W4021, Promega). Detection was performed using SuperSignal West Pico Chemiluminescent Substrate (Thermo Scientific) and the protein band sizes were quantified using Image J, normalized to GAPDH.

### Statistical analyses

For comparisons between three subsets, a one-way ANOVA was performed with Tukey’s Test to correct for multiple comparisons. All fluorescence-based values (PCR, flow cytometry, luminex data) were log-transformed before statistical analysis. For comparison between young and elderly data, an unpaired two-tailed Mann–Whitney test was performed.

## Electronic supplementary material

Supplememtary Figures
