# Supplementary material for: The pro-inflammatory phenotype of the human non-classical monocyte subset is attributed to senescence
Source: Cell Death Dis. 2018 Feb 15;9(3):266. doi: 10.1038/s41419-018-0327-1 (PMC5833376; doi:10.1038/s41419-018-0327-1)
Supplement: Supplementary file 1 — Supplememtary Figures [file 41419_2018_327_MOESM1_ESM.pdf]

Supplementary Figure S1

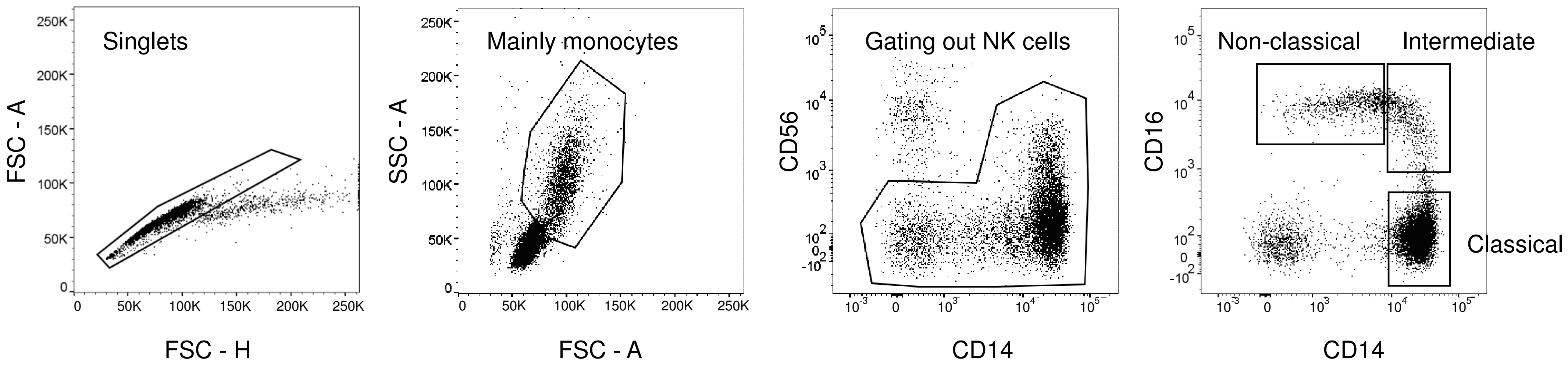

Flow cytometry gating strategy. Gating of total peripheral blood mononuclear cells (PBMCs) for analysis of the three monocyte subsets.

Supplementary Figure S2

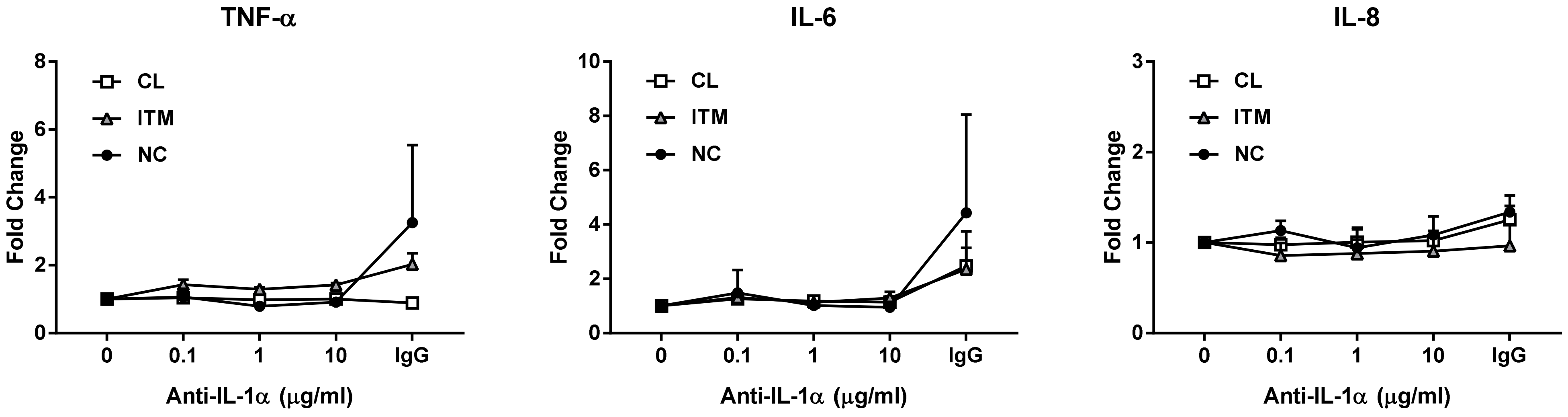

Treatment with anti-IL-1α does not inhibit SASP in non-classical monocytes. Fold change in secretion, with respect to no treatment, of TNF-α, IL-6 and IL-8 in the three monocyte subsets. Data represent the means ± SD; n = 3.

Supplementary Figure S3

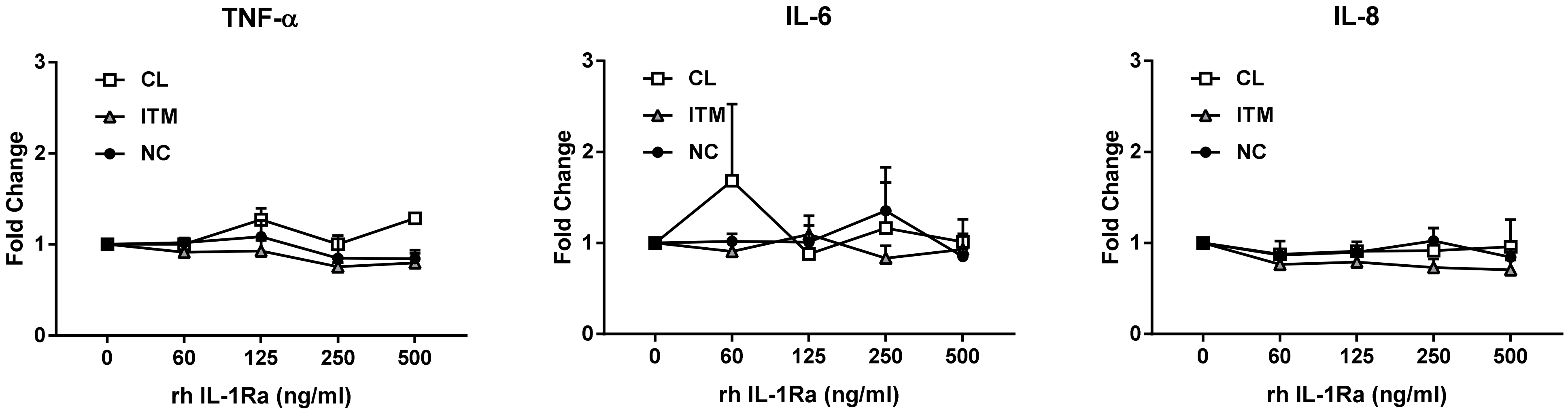

Treatment with rh IL-1Ra does not inhibit the SASP in non-classical monocytes. Fold change in secretion, with respect to no treatment, of TNF-α, IL-6 and IL-8 in the three monocyte subsets. Data represent the means ± SD; n = 3.

Supplementary Figure S4

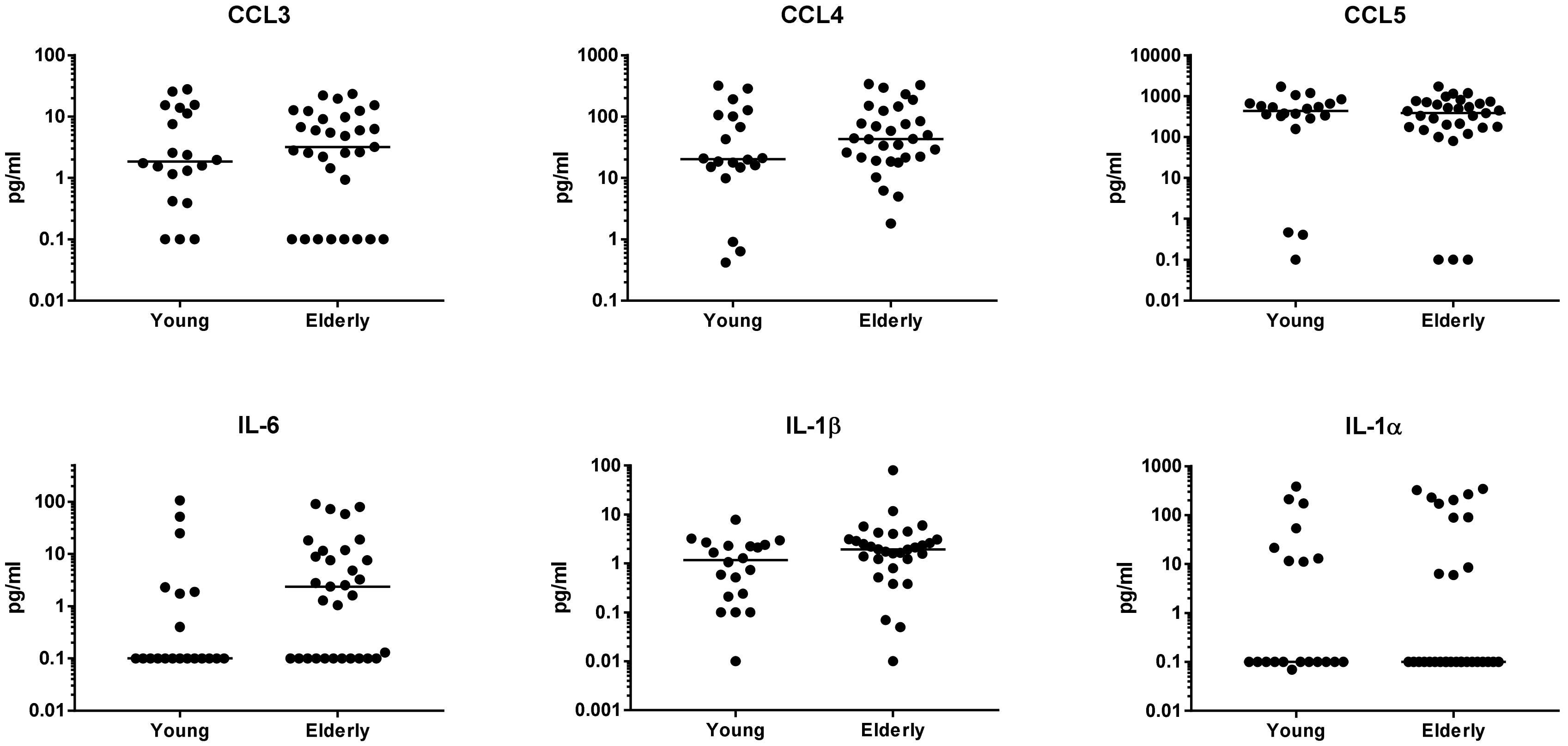

Plasma levels of cytokines are higher in the elderly. CCL3, CCL4, CCL5, IL-6 IL-1β and IL-1α levels in the plasma was analyzed by Luminex assay. Each dot represents one donor; line represents median; n = 20 for young, n = 30 for elderly.
